# Topical Carboxytherapy Modulates the Skin Microbiome Following CO_2_ Laser Resurfacing: A Pilot Study

**DOI:** 10.1111/jocd.70668

**Published:** 2026-01-12

**Authors:** Barbara Hernandez‐Rovira, Emma Villamaria, Julia Oh, Bengisu Ozarslan, Saranya Wyles

**Affiliations:** ^1^ Department of Dermatology Mayo Clinic Rochester Minnesota USA; ^2^ Universidad Central Caribe School of Medicine Bayamon Puerto Rico; ^3^ Duke University Durham North Carolina USA

**Keywords:** CO_2_ laser resurfacing, cutibacterium acnes, facial rejuvenation, post‐treatment microbiome, skin microbiome, topical carboxytherapy, wound healing

To the Editor,

Skin microbiome contributes to cutaneous homeostasis and barrier defense by regulating keratinocyte proliferation, angiogenesis, and immune responses [1, 2]. Carbon dioxide (CO_2_) laser resurfacing, widely used for photoaging and scar revision, induces controlled dermal injury and transient skin barrier disruption to trigger the wound healing cascade. Topical carboxytherapy, which delivers CO_2_ transdermally, is proposed to enhance tissue regeneration by improving oxygenation and microvascular perfusion [3]; however, its mechanistic impact on skin physiology and microbial communities remains poorly defined. Given the role of skin microbiota in wound healing and inflammation, understanding how these procedures affect microbial dynamics is clinically relevant. This pilot study aimed to characterize the facial skin microbiome following full‐face CO_2_ fractional resurfacing and post‐procedural treatment with topical carboxytherapy (CO_2_ Lift, Lumisque, Weston, FL).

Two participants were enrolled and assigned to one of two post‐laser care groups: the placebo group (*n* = 1) received standard care with a petroleum‐based ointment, while the intervention group (*n* = 1) was treated with a gel‐formulated carboxytherapy. VISIA‐CR (Canfield Scientific Inc., Parsippany, NJ) imaging was performed at baseline and 12 weeks post‐procedure (Figure 1). Microbiome samples were collected from the left versus right malar cheek at baseline and week 4 using standardized swabbing. Microbial profiling was performed via 16S rRNA gene sequencing with genus‐level taxonomic assignment; sequencing depth and diversity metrics were evaluated descriptively, without statistical testing, due to the limited sample size. Genus‐level taxonomic composition is shown in Figure 2. Given that only one participant was included per group, this exploratory pilot study was not powered for statistical comparison between groups, and all findings are presented as hypothesis‐generating observations without inference of group differences or treatment effects.

**FIGURE 1:**
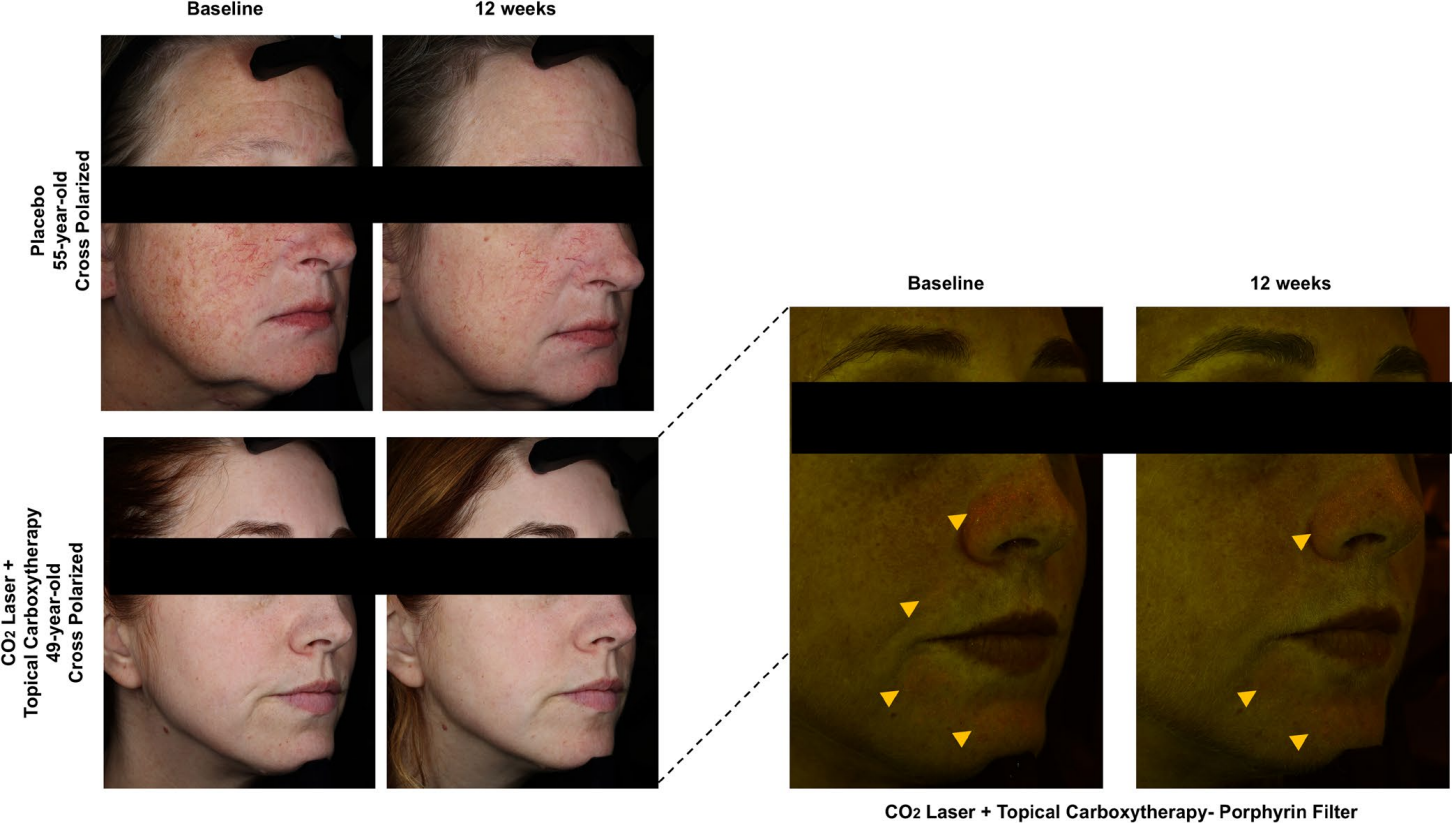
Facial skin imaging before and after fractional CO_2_ laser resurfacing and topical carboxytherapy. VISIA‐CR imaging captured at baseline and 12 weeks post‐treatment, demonstrating cutaneous changes in participants receiving either placebo or topical carboxytherapy following fractional CO_2_ laser resurfacing. Top row: Cross‐polarized images from the placebo group. Bottom row: Cross‐polarized and porphyrin‐filter images from the topical carboxytherapy treatment arm, showing follicular porphyrin signal in the nasolabial folds, mental fold, and chin region.

**FIGURE 2:**
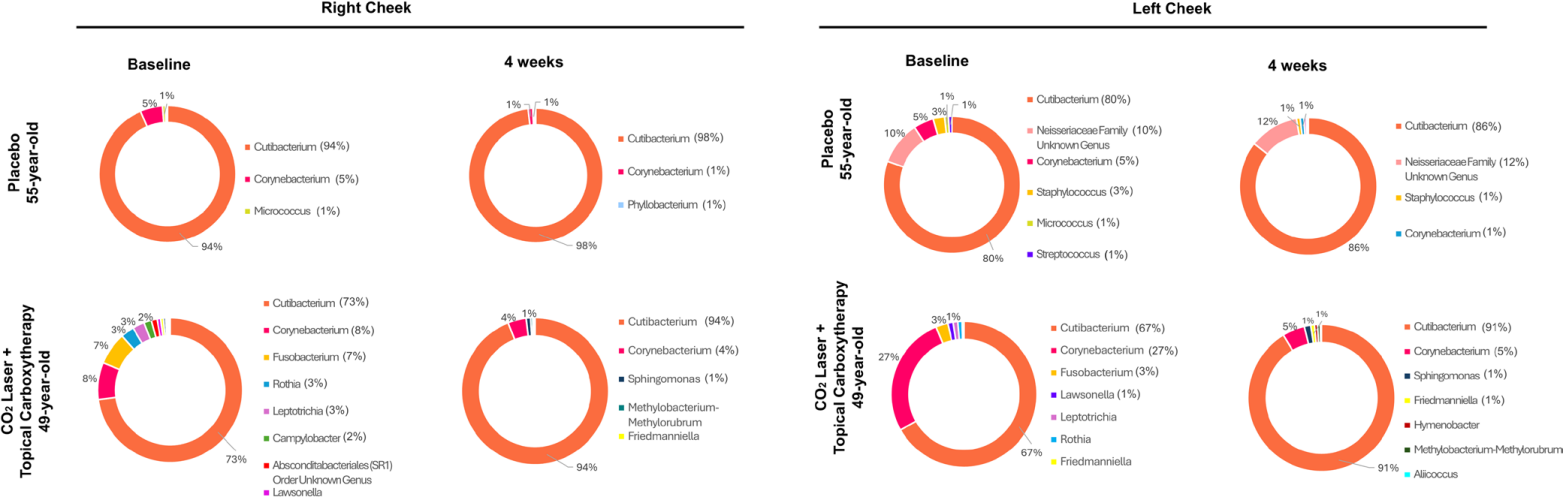
Facial skin microbiome composition before and after fractional CO_2_ laser resurfacing and topical carboxytherapy. Genus‐level relative abundance based on observed amplicon sequence variants (ASVs) from the right and left cheek at baseline and 4 weeks post‐treatment. The skin microbiome was consistently dominated by *Cutibacterium* spp. and *Corynebacterium* spp. across both pre‐ and post‐treatment timepoints. Topical carboxytherapy after laser resurfacing was associated with a greater post‐treatment increase in *Cutibacterium* spp. abundance. Top row: Placebo group. Bottom row: Topical carboxytherapy treatment arm.

*Cutibacterium* spp. was the dominant genus across all subjects, with bilateral detection at both time points. Relative abundance increased from 79% at baseline to 92% at week 4. *Corynebacterium* spp. was the second most abundant genus (11% at baseline, 3% at week 4), followed by low‐abundance taxa including *Streptococcus* spp. and *Staphylococcus* spp. Alpha diversity patterns varied between participants at baseline and over time; both groups exhibited a slight decline. These findings are reported descriptively only. Beta diversity analysis suggested overall compositional stability within individuals between baseline and follow‐up, though no formal clustering or between‐group comparisons can be inferred.

An observed increase in the relative abundance of *Cutibacterium* spp., presumed to be *C. acnes*, following CO_2_ laser resurfacing may suggest a potential role in maintaining microbial balance and supporting tissue repair. As the primary commensal of sebaceous skin, *C. acnes* is well‐adapted to lipid‐rich environments and contributes to skin homeostasis through immunomodulation, lipid metabolism, and inhibition of opportunistic pathogens [4]. Reduced *C. acnes* diversity has been associated with aging and frailty, while its antioxidant activity and ability to suppress biofilm formation support a protective role in wound healing [1, 5]. These mechanistic interpretations are intended to contextualize the observed microbiome patterns rather than establish causality.

Although limited by a small sample size, these findings indicate that neither CO_2_ laser resurfacing nor adjunctive topical carboxytherapy disrupts the composition or diversity of the facial skin microbiome. Given the bidirectional interaction between skin microbiota and wound regeneration, future studies should include larger cohorts and metagenomic sequencing to further define the impact of adjunctive treatments on microbial dynamics. Nonetheless, this pilot study suggests post‐procedural microbiological stability and highlights the feasibility of incorporating microbiome endpoints in studies aimed at optimizing dermatologic interventions.

## Author Contributions

Barbara Hernandez‐Rovira: Writing‐original draft (equal); formal analysis (lead); visualization. Emma Villamaria: Writing‐original draft (equal); formal analysis (supporting). Julia Oh: Writing‐review and editing (equal); validation. Bengisu Ozarslan: Writing‐review and editing (equal); formal analysis (supporting). Saranya Wyles: Conceptualization; methodology; supervision; writing‐review and editing (equal).

## Funding

This study was supported by Lumisque Skincare, LLC. The funder was not involved in study and manuscript preparation.

## Disclosure

AI was not used in the composition of this manuscript.

## Ethics Statement

This study was approved by the Mayo Clinic Institutional Review Board (IRB 23‐005182).

## Consent

Written informed consent was obtained from all participants included in this study.

## Conflicts of Interest

The authors declare no conflicts of interest.

## Data Availability

The data that support the findings of this study are available from the corresponding author upon reasonable request.
